# Optical coherence tomography–enabled classification of the human venoatrial junction

**DOI:** 10.1117/1.JBO.30.1.016005

**Published:** 2025-01-21

**Authors:** Arielle S. Joasil, Aidan M. Therien, Christine P. Hendon

**Affiliations:** Columbia University, Department of Electrical Engineering, New York, United States

**Keywords:** optical coherence tomography, radiofrequency ablation, atrial fibrillation, machine learning, deep learning

## Abstract

**Significance:**

Radiofrequency ablation to treat atrial fibrillation (AF) involves isolating the pulmonary vein from the left atria to prevent AF from occurring. However, creating ablation lesions within the pulmonary veins can cause adverse complications.

**Aim:**

We propose automated classification algorithms to classify optical coherence tomography (OCT) volumes of human venoatrial junctions.

**Approach:**

A dataset of comprehensive OCT volumes of 26 venoatrial junctions was used for this study. Texture, statistical, and optical features were extracted from OCT patches. Patches were classified as a left atrium or pulmonary vein using random forest (RF), logistic regression (LR), and convolutional neural networks (CNNs). The features were inputs into the RF and LR classifiers. The inputs to the CNNs included: (1) patches and (2) an ensemble of patches and patch-derived features.

**Results:**

Utilizing a sevenfold cross-validation, the patch-only CNN balances sensitivity and specificity best, with an area under the receiver operating characteristic (AUROC) curve of 0.84±0.109 across the test sets. RF is more sensitive than LR, with an AUROC curve of 0.78±0.102.

**Conclusions:**

Cardiac tissues can be identified in benchtop OCT images by automated analysis. Extending this analysis to data obtained *in vivo* is required to tune automated analysis further. Performing this classification *in vivo* could aid doctors in identifying substrates of interest and treating AF.

## 1 Introduction

Atrial fibrillation (AF) is the most common arrhythmia, affecting at least 2.3 million people in the United States. If left untreated, serious health complications can occur, including cardiac arrest and stroke.^1^^–^^3^ Doctors may prescribe pharmaceutical interventions to return the heart to sinus rhythm, but the patient may require catheter radiofrequency ablation (RFA) if medications do not alleviate symptoms. Since the discovery that the ectopic heartbeats that cause AF originate from the pulmonary veins (PVs),^4^ pulmonary vein isolation (PVI) has become the most common approach to treating paroxysmal AF with RFA.^5^ This procedure aims to place circumferential lesions outside the PV ostia to prevent impulses from reaching the left atrium and initiating AF. This procedure is not always successful the first time as AF may reoccur due to non-transmural lesions, gaps between the lesions, and electrical reconnection.^6^ PV stenosis is one of the risks associated with RFA. With the improvement of techniques, the number of patients experiencing PV stenosis post-RFA is ∼3%.^7^ This condition can be deadly if left untreated but is challenging to detect due to non-specific symptoms that may not present until weeks or months after the procedure.^7^^,^^8^ Other injuries sustained from RFA include perforation, dissection, and PV thrombosis.^8^

Doctors perform RFA with real-time guidance from the catheter (tissue impedance, temperature, contact force, etc.) and via fluoroscopy. Indirect feedback from the catheter can provide information that is helpful for guiding ablation. Although these techniques can visualize the region of interest, fluoroscopy requires ionizing radiation,^9^^,^^10^ and all of these techniques have low resolution. Direct visualization of tissue during PVI procedures would enhance the doctors’ ability to deliver effective ablation without injuring the patient.

Optical coherence tomography (OCT) is a volumetric, non-invasive, optical imaging modality with micron resolution, capable of imaging tissue 1- to 2-mm deep. With these features, OCT has been demonstrated to provide structural information on the endocardium, myocardium, and epicardium, including myofiber orientation.^11^^–^^16^ Deep learning analysis of OCT images has been shown to have utility in analyzing various human tissues, including the retina,^17^^,^^18^ cornea,^19^ brain,^20^ bone,^21^ vasculature,^22^ and heart.^23^^,^^24^ The addition of polarization contrast has been demonstrated to enable the characterization of myocardial fiber architecture within small animal models^25^^–^^29^ and the assessment of fibrosis.^30^ Real-time *in vivo* and *in vitro* monitoring of RFA has been performed on evaluating lesion formation, substrate detection, and catheter contact,^23^^–^^25^^,^^31^^–^^35^ in large animals and humans. Other modalities, such as near-infrared spectroscopy and multi-spectral endoscopy,^36^^–^^39^ have been evaluated to monitor RFA lesion formation and lesion transmurality.

Imaging the PV and LA junction, or venoatrial junction, with OCT intraoperatively would ensure that the PV is appropriately isolated. In this work, we aim to identify OCT features to distinguish the left atrium and pulmonary vein, and we propose machine and deep learning algorithms to evaluate the potential of algorithmic guidance in real time. Distinguishing the LA from the PV is imperative to ensure that PVI is performed correctly to prevent pulmonary vein stenosis and to guide ablation energy delivery.

## 2 Methods

### 2.1 Experimental Samples

**Fig. 1:**
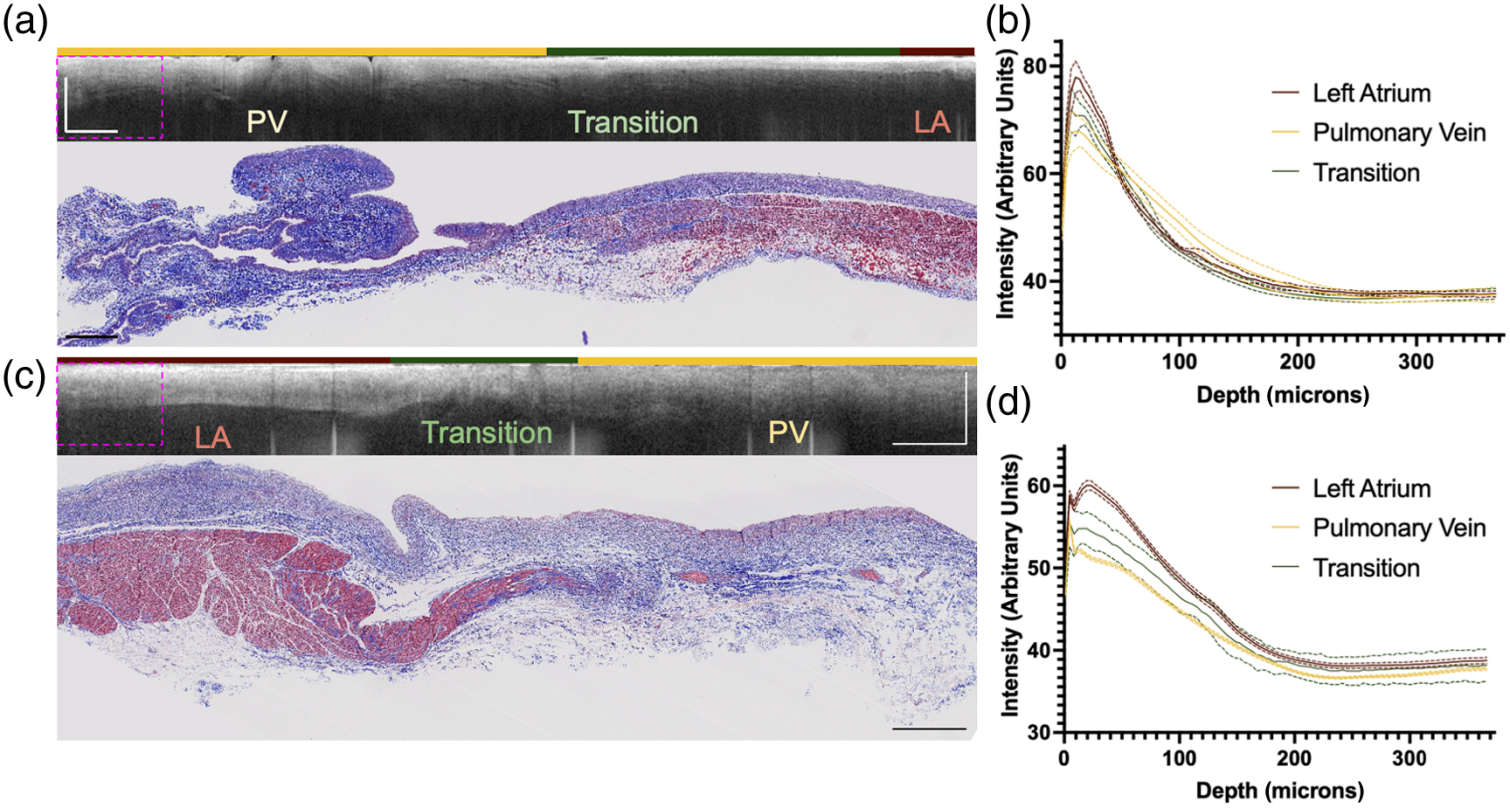
Examples of B-scans, corresponding histology, and figures showcasing the intensity as a function of depth from the volumes in the dataset are shown. The dashed, pink boxes in the B-scans are 367  μm×800  μm. (a) This B-scan and corresponding Masson’s trichrome image are from donor 4. The venous tissue of the PV allows for deeper penetration of light. The endocardium is more scattering. (b) This figure showcases how the intensity of light decays with depth. The patches used in this analysis had the dimension of the dashed, pink boxes in the B-scans (∼15.3  mm×3.3  mm). The A-lines in these patches were averaged. No patches with multiple labels were used. In this volume, the light intensity decays more slowly in the pulmonary vein than in the left atrium. (c) This Masson’s trichrome image and B-scan are from donor 10. (d) In this sample, the light intensity in the pulmonary vein decays faster than that of the left atrium. All scale bars represent 1 mm.

The OCT dataset used throughout this work consists of comprehensive imaging of 26 venoatrial junctions from 10 diseased, post-mortem hearts from a previous study.^40^ The demographics and clinical history of the donors are described in Table 1. All samples were de-identified, received from the National Disease Research Interchange (NDRI), and considered non-human subject research by the Columbia University Institutional Review Board (IRB). The venoatrial junctions were recovered within 24 h after death and imaged while submerged in phosphate-buffered saline using the TELESTO I (Thorlabs GmbH, Dachau, Germany) spectral-domain (SD) OCT system. The samples were imaged and flattened using a Thorlabs OCT IMM-3 immersion style sample z-Spacer while in phosphate-buffered saline. The system had a lateral resolution of 15  μm, an axial resolution of 6.5  μm, and an imaging depth of 2.51 mm in air. It had a center wavelength of 1325 nm with a bandwidth of 150 nm. Stitching overlapping individual volumes allowed for comprehensive imaging of the venoatrial junctions.^40^^,^^41^ The stitched OCT volumes were labeled by a laboratory member who was not involved in algorithm development with the guidance of Masson Trichrome histopathology as the gold standard. The classes in this dataset are LA, transition tissue (a combination of LA and PV), and PV. The transition region begins at the first appearance of venous features within the left atrial myocardium and ends where only adventitia and venous media of the pulmonary vein are found. Sample B-scans and corresponding Masson’s trichrome images are shown in Fig. 1.

**Table 1:** Cardiovascular disease history of human donors and the number of venoatrial junctions extracted per donor heart. AF, atrial fibrillation; CAD, coronary artery disease; CHF, congestive heart failure; HLD, hyperlipidemia; HTN, hypertension; MI, myocardial infarction; PVD, peripheral vascular disease; VHD, valvular heart disease.

Heart ID	Age	Sex	Cardiovascular disease history	Number of venoatrial junctions
1	77	F	CAD, HTN, CHF, AF, PVD	4
2	70	F	CHF, HTN	4
3	46	F	HTN, CAD, MI	1
4	67	M	MI, HTN, HLD	3
5	59	F	HTN	3
6	67	M	CHF, VHD, HLD	3
7	58	M	CAD, CHF, HTN, HLD	4
8	68	M	HTN, CAD, HLD	2
9	62	F	HLD, CAD	1
10	58	F	VHD	1

Patches extracted from the stitched B-scans of the volumes were used for analysis. Before extraction, the tissue was digitally flattened. Various sizes of OCT image patches were extracted to examine the effect of patch size on classification. Non-overlapping candidate patch dimensions were 118  μm (depth) × 224  μm (width), 118  μm (depth) × 800  μm (width), 235  μm (depth) × 224  μm (width), and 235  μm (depth) × 800  μm (width). Analysis presented in this work was ultimately conducted on non-overlapping patches with a dimension of 235  μm×800  μm, (64  pixels×128  pixels). For analysis, only PV and LA patches were used for model development and testing as the transition region contained characteristics of both tissue types. OCT image patches must also have the following characteristics: (1) 90% of image pixels must have an intensity greater than zero, (2) all A-lines within the patch have the same label (i.e., only LA or PV), and (3) there are no 0 intensity A-lines in the patch. Due to the intensity signal fall-off in OCT images, only the top 235  μm (64 pixels) of the B-scans were considered for analysis. Although the patient population (10) and the sample size (26) are limited, the venoatrial junctions were comprehensively acquired, allowing for the extraction of many OCT patches. The stitched volumes within our dataset had average dimensions of 16.7  mm (x)×8.7  mm (y), with standard deviations of 7.1  mm (x)×3.3  mm (y). A total of 38,784 patches were extracted; 78% of the patches were LA, and 22% were PV. Stratification by venoatrial junction led to a variation of total patches and distribution of each class within the train, validation, and test sets across folds. Patches from each junction were kept exclusively in the test, train, or validation set.

### 2.2 Feature Engineering

We performed statistical analysis to determine the discriminatory power of each patch-derived feature. Unpaired t-tests using Welch’s correction were performed on the average feature values across the 26 samples and 10 donors using GraphPad Prism 10.2.3 (Dotmatics, Boston, Massachusetts, United States). P-values less than 0.05 were considered statistically significant. We performed random forest (RF) classification across a sevenfold cross-validation (CV) scheme to determine what features best distinguished PV and LA patches. Receiver operating characteristic (ROC) curve analysis was performed on statistically derived patch features to determine further how well individual features discriminate between LA and PV. R (version 4.3.1) was used to find the Pearson correlation coefficients among all variables. The texture, statistical, and optical features extracted from these patches are presented in Table 2. Entropy, kurtosis, and mean were not considered during the feature engineering task due to the area under the ROC curve (AUROC) of <0.6. The attenuation coefficient was calculated using the method described in Ref. 42. The combination of Pearson’s correlation coefficient, random forest feature importance, area under the ROC curve, and results from t-tests with Welch’s correction informed the selected features. Features with ρ<−0.7 or ρ>0.7 were less suited for the classification task.

**Table 2:** Features extracted from the patches in order of decreasing area under the ROC curve.

Feature	Description
Correlation (texture)	Gray level co-occurrence matrix (GLCM) correlation
Attenuation coefficient standard deviation (optical)	Standard deviation of the attenuation coefficient map
Variance (statistical)	Variance of the pixels
Energy (texture)	GLCM energy
Skewness (statistical)	Skewness of the pixels
Contrast (texture)	GLCM contrast
Homogeneity (texture)	GLCM homogeneity
Attenuation coefficient (optical)	Attenuation coefficient
Entropy (statistical)	Entropy of the pixels
Kurtosis (statistical)	Kurtosis of the pixels
Mean (statistical)	Mean of the pixels

### 2.3 Automated Classification Approaches

**Fig. 2:**
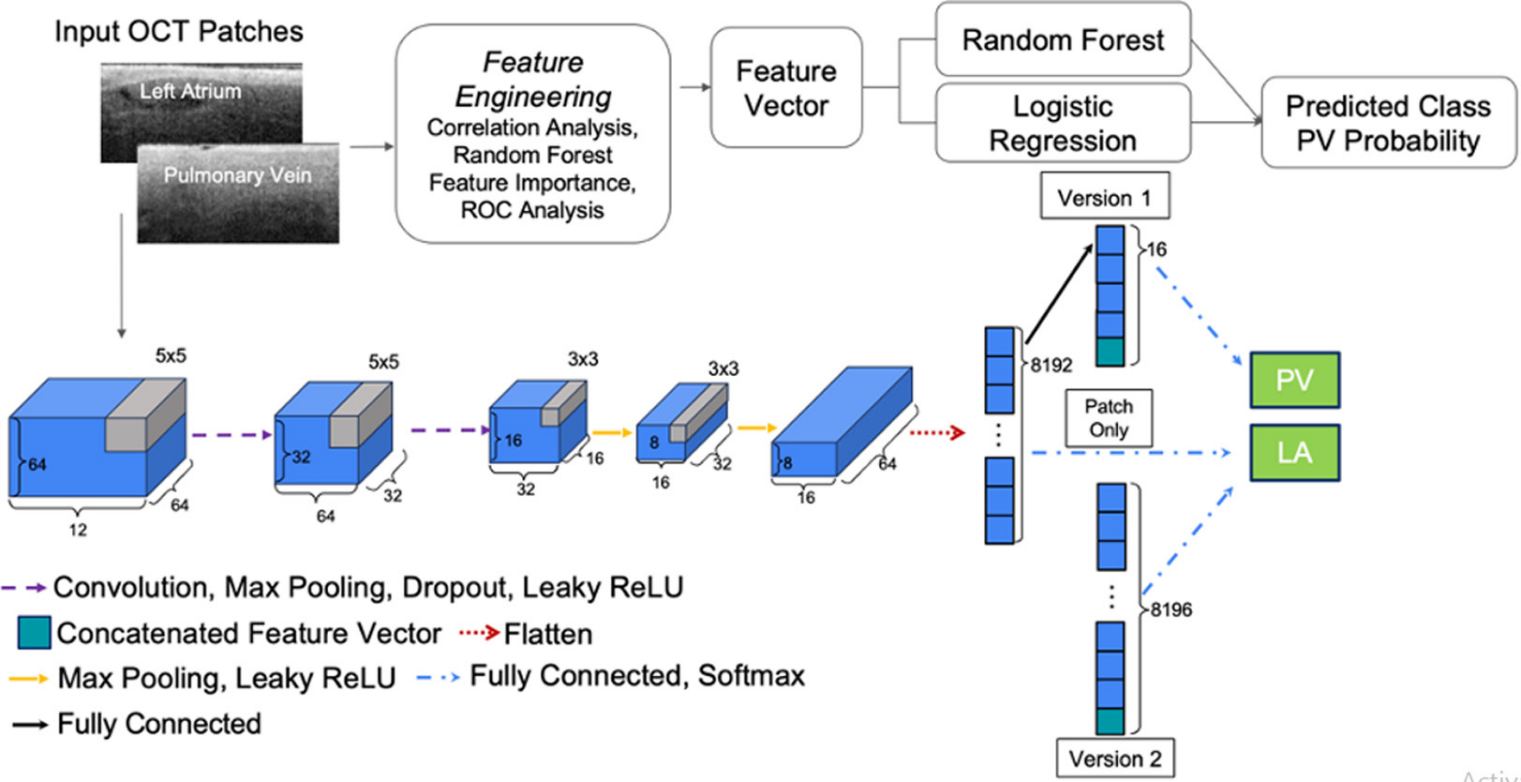
Algorithm flowchart for both the machine learning and deep learning approaches. Features selected based on feature engineering techniques were input into the machine learning algorithms. Depending on the implementation, deep learning approaches took only the patches or a combination of patches and selected features as input.

Engineered features and OCT image patches were used as input to different classification algorithms to evaluate performance. The algorithmic pipeline is shown in Fig. 2. All venoatrial junctions were evaluated as part of the test set in a sevenfold CV scheme to ensure that each junction appeared in the test set only once. For the first sixfolds, the training, validation, and testing split had 18, 4, and 4 junctions, respectively. To avoid repetition of any samples in the test set, the last fold featured a training, validation, and testing split of 20, 4, and 2 junctions, respectively.

#### 2.3.1 Machine learning approach

We implemented logistic regression (LR) and random forest (RF) as the machine learning algorithms. The classifiers were deployed using scikit-learn (version 1.2.0) using Python 3.9.7. For both algorithms, the training and validation features were combined to create a 20-sample (splits 1 to 6) or 22-sample (split 7) training sets. LR was implemented with $l_{2}$ regularization with a penalty coefficient of 1.0 to prevent overfitting. Before performing classification with LR, features were standardized by rescaling to zero mean and unit variance. The mean and standard deviation of the training features were used to transform the test set. The RF implementation used 100 trees and the Gini criterion. The selected features were not scaled before classification.

#### 2.3.2 CNN ensemble approach

CNN architectures were also developed to perform the OCT patch classification. The networks took images and engineered features as inputs by concatenating features before the final dense activation. The feature data provided to the network were rescaled to have zero mean and unit variance in the same way as the machine learning approach. Input image intensity values were mapped from unsigned 8-bit integers to floating point numbers in the interval [0, 1]. Dataset augmentation was also performed by adding a 50% chance of flipping the image across the vertical axis. The network performance was optimized by adjusting network architecture and size, hyperparameter tuning (learning rate = 0.0001, dropout probability = 0.15, batch size = 256), experimenting with the optimizer (Adam, SGD, SGD with momentum, AdaGrad), and class balancing adjustments to the loss function (cross entropy and class weighting). The overall plain CNN architecture consisted of four convolutional layers, some containing dropout, followed by a fully connected network from an 8192 convolutional parameter embedding down to the predicted class probability. Two variations of the plain CNN, version 1 (V1) and version 2 (V2), were also tested. The V1 and V2 networks were devised to examine the effect of introducing the engineered features at different network layers, resulting in variable emphasis on the engineered features. Effectively, the V1 architecture had a greater parameter count and emphasized the engineered features more than V2. The V2 architecture concatenated in the top 4 features before the dense layer, increasing the parameter count in the final dense layer to 8196. The V1 architecture had an additional dense layer resulting in 12 parameters, the four top-engineered features were then concatenated, and the last dense activation took the 16 parameters as input and yielded the class probability. The deep learning algorithms were implemented in PyTorch (version 2.1.2). Gradient class activation mapping (GradCAM)^43^ was used to assess which image regions contributed most to classification.

### 2.4 Evaluation Metrics

Accuracy, sensitivity, specificity, Matthews correlation coefficient (MCC), and the area under the receiver operating characteristic curve (AUROC) were measured for each test split. The MCC^44^ ranges between −1 and 1, but the metric was normalized between 0 and 1 in this study for comparison with the other metrics. The MCC is defined in Eq. (1),

$$\mathrm{MCC}=\frac{\mathrm{TP}\cdot \mathrm{TN}-\mathrm{FP}\cdot \mathrm{FN}}{\sqrt{\left(\mathrm{TP}+\mathrm{FP})\cdot (\mathrm{TP}+\mathrm{FN})\cdot (\mathrm{TN}+\mathrm{FP})\cdot (\mathrm{TN}+\mathrm{FN}\right)}}.$$ (1)

The normalized MCC is calculated as $\text{normMCC}=\frac{\mathrm{MCC}+1}{2}$.

## 3 Results and Discussion

### 3.1 Feature Selection and Patch Size

Within the LA, the endocardium appears as a highly scattering layer, which is followed by the less intense myocardium. The endocardial thickness was observed to vary spatially within the left atrium and among donors. The PV has different appearances in OCT images. Within the PV, the light penetrates deeper, does not contain the highly scattering endocardial layer, and has lower attenuation. This trend is highlighted in Fig. 1.

**Fig. 3:**
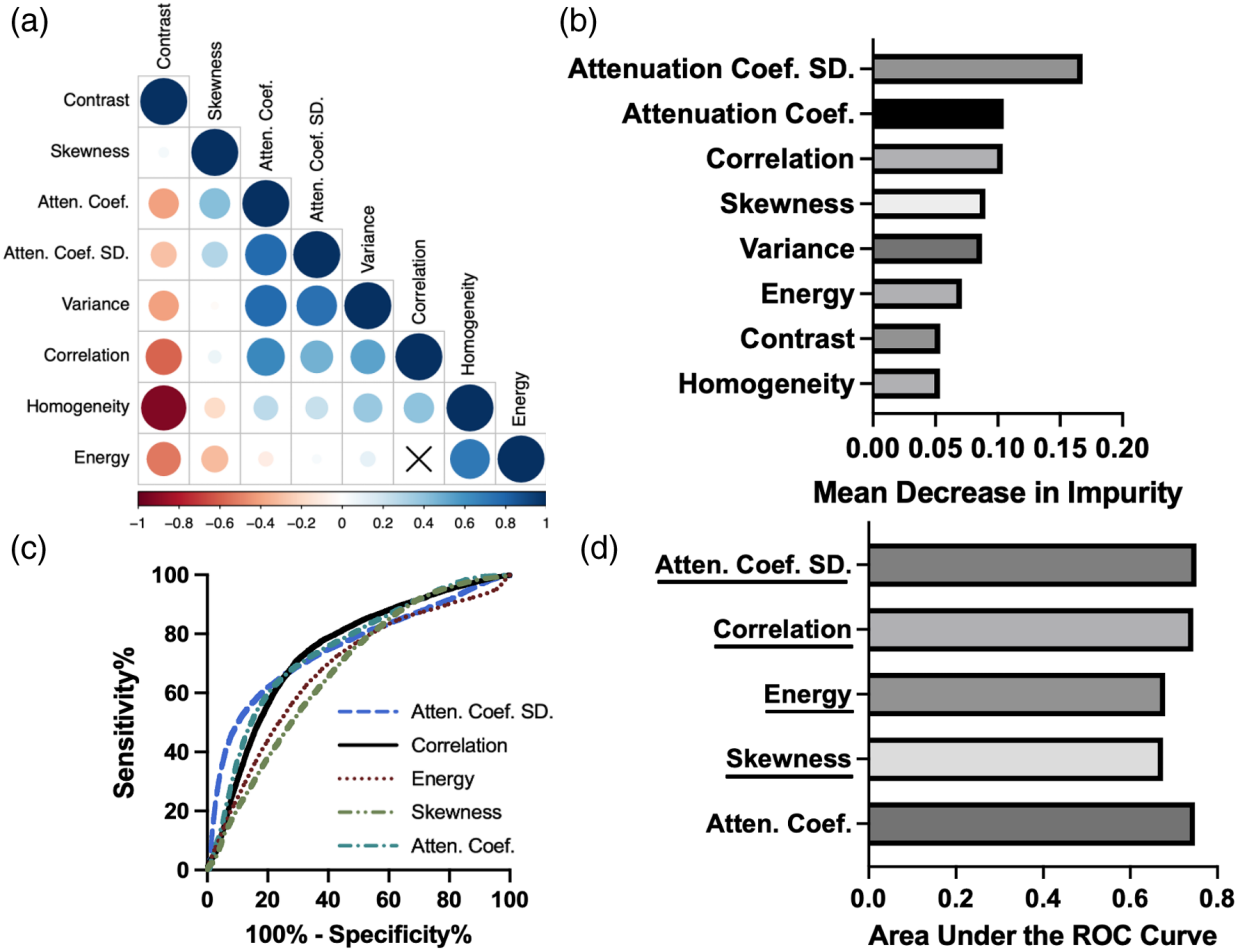
Feature engineering strategy to select the features for input into classification algorithms. (a) Ten features were extracted, and the correlation matrix of the features is shown. Locations with “x” are indicative of a statistically insignificant correlation (P>0.05). The size and hue of the circles describe the magnitude of the correlation. (b) The feature importance of RF as a function of the mean decrease in impurity averaged across seven folds. The attenuation coefficient SD is the most important, whereas homogeneity is the least important feature. (c), (d) The ROC curves and the area under the ROC curves of the top features and the attenuation coefficient. All of these features have an area under the ROC curve over 0.6, indicating discriminating power. The final selected features were correlation, skewness, attenuation coefficient SD, and energy.

Based on feature engineering, we determined the top features to be correlation, attenuation coefficient SD, energy, and skewness. The mean values are shown in Fig. 3(b). As variance and attenuation coefficient SD were highly correlated with ρ≥0.7, we did not use variance in the final algorithm. ROC analysis and RF importance align as these features have the highest AUROC, as seen in Figs. 3(c) and 3(d). We investigated the impact of the attenuation coefficient as it is closely related to the attenuation coefficient SD.

**Fig. 4:**
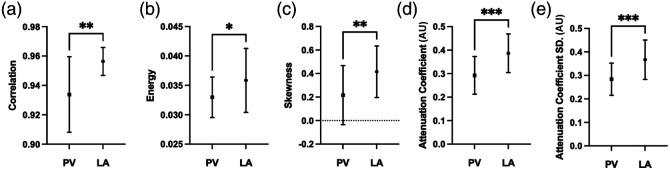
Distribution of average feature values across venoatrial junctions, N=26, for the 235  μm×800  μm patches. The line represents the mean ± standard deviation. T-testing indicates that the means are significantly different. * indicates P≤0.05, ** represents P≤0.01, and *** represents P≤0.001.

The distributions of the features highlighted in Fig. 3(d) are shown in Fig. 4. The means of the averaged features across the venoatrial junctions are significantly different. Among the junctions, we find that the LA has a higher attenuation coefficient than the PV, with sample average attenuation coefficients of 0.6042 and $0.5227\;{\mathrm{mm}}^{-1}$, respectively, as seen in Fig. 4. The attenuation coefficient and the attenuation coefficient SD distinguish the LA and PV as the LA is more scattering and layered than the PV, which is reflected in the distributions of both features in Fig. 4. On average, both features are higher within the LA than the PV.

We found that non-overlapping patches of size 235  μm×800  μm yielded the best results based on metrics presented in Table 3. Of these sizes, the depth of 235  μm showcased the difference in signal intensity roll-off between the PV and LA the best, as seen in Fig. 1. Patches containing a lot of background disrupted the feature extraction. Classifying the smaller 235  μm×224  μm patches with RF and LR required all of the features in Table 2 to obtain results similar to those reported for the larger patches in Table 3.

**Table 3:** Mean ± standard deviation of the metrics calculated across the seven folds for all methods of the top features extracted from the 235  μm×800  μm patches at a PV classification threshold of 0.5.

	Machine learning: logistic regression	Machine learning: random forest	Deep learning: patches only	Deep learning: patches + features version 1	Deep learning: patches + features version 2
Accuracy	0.78 ± 0.071	0.79 ± 0.072	0.740 ± 0.116	0.710 ± 0.116	0.730 ± 0.119
Sensitivity	0.29 ± 0.215	0.460 ± 0.206	0.79 ± 0.170	0.76 ± 0.178	0.75 ± 0.190
Specificity	0.93 ± 0.059	0.88 ± 0.091	0.71 ± 0.165	0.680 ± 0.198	0.710 ± 0.177
MCC	0.64 ± 0.110	0.69 ± 0.089	0.73 ± 0.083	0.70 ± 0.079	0.714 ± 0.082
AUROC	0.80 ± 0.106	0.78 ± 0.102	0.840 ± 0.109	0.82 ± 0.136	0.82 ± 0.118

### 3.2 Classification Performance

**Fig. 5:**
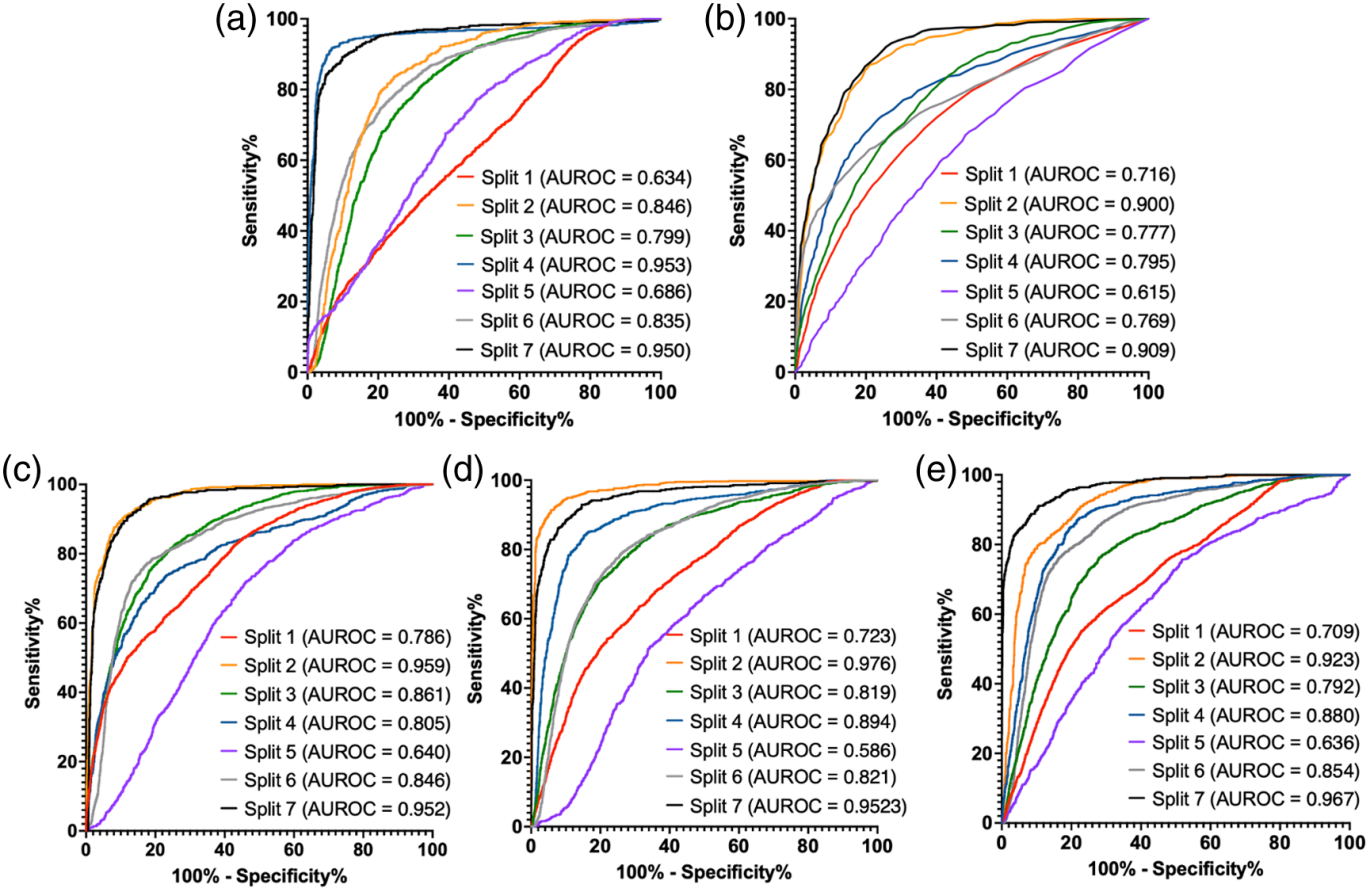
ROC curves across the sevenfold test set for all methods: (a) logistic regression (machine learning), (b) random forest (machine learning), (c) patch-only CNN (deep learning), (d) ensemble CNN V1 (deep learning), and (e) ensemble CNN V2. Splits 1 to 6 had 4 PV-LA junctions in the test set, whereas split 7 had only two samples of PV-LA junctions in the test set to avoid the repetition of samples. Classification performance varied depending on the implementation; however, all algorithms performed best on splits 2 and 7 and had the worst performance on split 5. AUROC, area under the receiver operating characteristic curve.

**Fig. 6:**
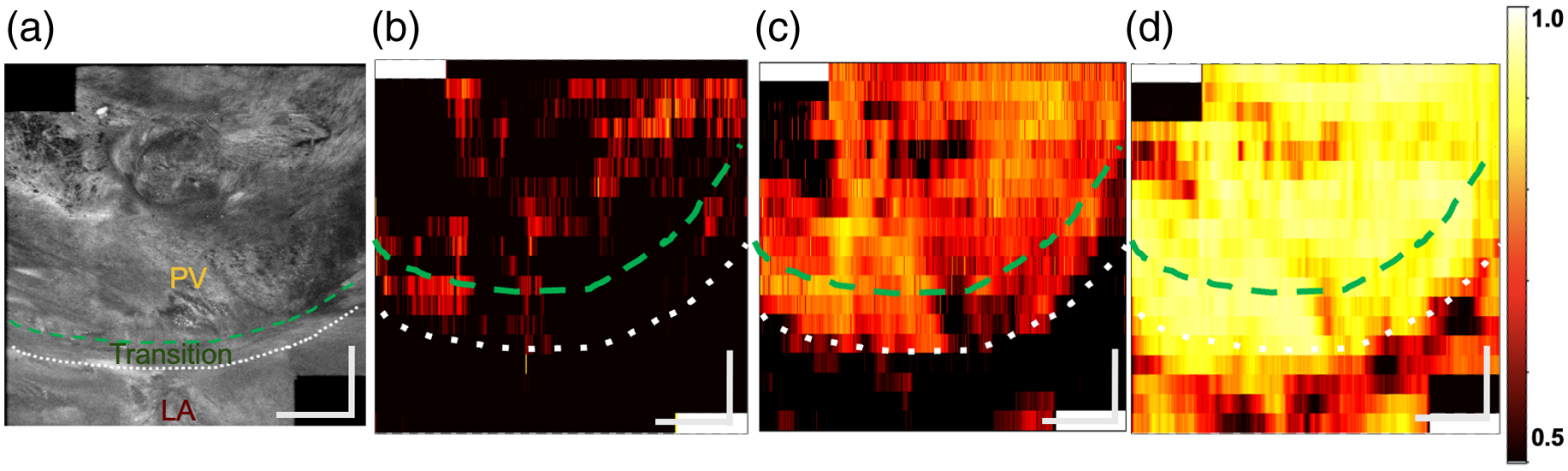
*En face* image (a) and probability maps for the methods: (b) RF (machine learning), (c) patch-only CNN (deep learning), and (d) ensemble CNN V1 (deep learning) of a sample from donor 4 (split 2). The ensemble CNN deep learning version 1 (d) classified the PV most confidently of all the algorithms. The CNN architecture that took patches as input also performed well, but the class probabilities across the volume were not as high. Samples with prediction probabilities of 0.5 and higher are classified as PV. The color bar is the probability of PV classification. The AUROC curve for CNN ensemble version 1 for this volume is 97.5%. The scale bars are 1 mm.

**Fig. 7:**
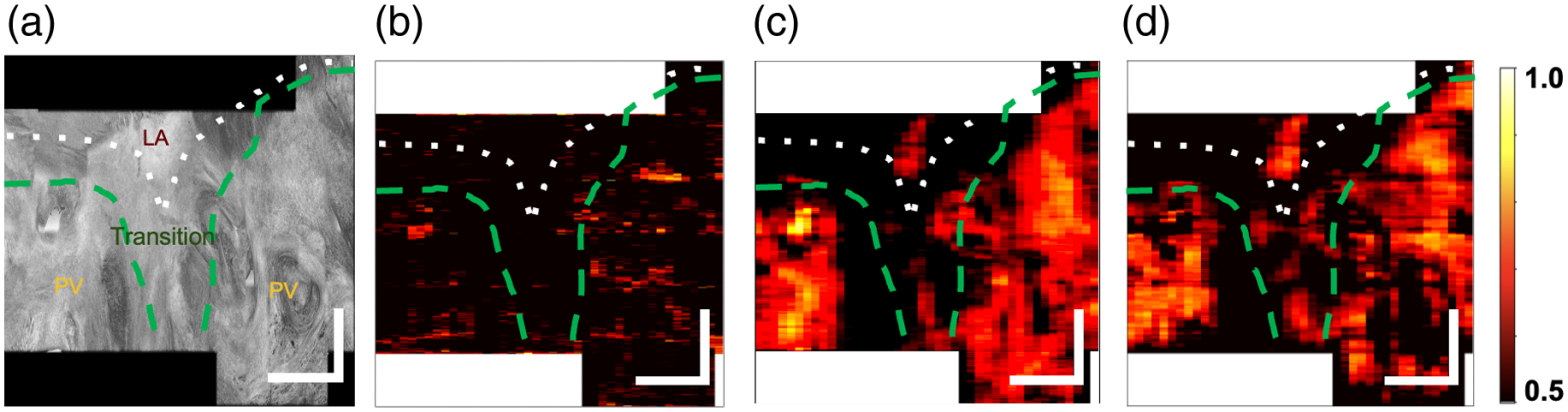
*En face* image (a) and probability maps for the methods: (b) RF (machine learning), (c) patch-only CNN (deep learning), and (d) ensemble CNN V1 (deep learning) from donor 4 (split 6). The CNN with patches only (c) and the ensemble CNN (d) classified the PV most confidently. The patches provide the CNNs additional contrast that random forest does not have, leading to fewer positive predictions. In these maps, the patch CNN (c) classifies fewer LA patches as PV than the ensemble CNN that takes patches as input (d). The color bar is the probability of PV classification. Samples with prediction probabilities of 0.5 and higher are classified as PV. The AUROC curve for CNN ensemble version 1 for this volume is 74.8%. The scale bars are 1 mm.

Figure 5 and Table 3 showcase the classification performance of the classification models. The DL patches only, V1 architecture, and V2 architecture perform very similarly; it is difficult to identify a clear winner from the summary statistics. Of the ML algorithms, RF was more sensitive than LR at the expense of specificity. RF is more flexible as it is non-parametric and does not make assumptions about the distributions of the data in contrast to LR. The ensemble DL V1 and V2 CNN methods used the top-engineered features and features extracted by the CNN to aid in its classification. Despite the added information and associated increased network parameter count, it is unclear whether the ensemble classification networks perform better than the more simple patch-based network. In this context, we believe that the patch-based CNN is the best-performing network as it has almost indistinguishable results from the DL V1 and V2 networks with fewer parameters. CNNs have dramatically increased parameter count and computational complexity to LR and RF and may be capable of extracting greater statistical contrast than the engineered features. Figures 6 and 7 show probability maps of PV classification of venoatrial junctions from split 2 and split 6, respectively. PV was classified exceptionally in Fig. 6, whereas Fig. 7 features a venoatrial junction that is classified as expected.

**Fig. 8:**
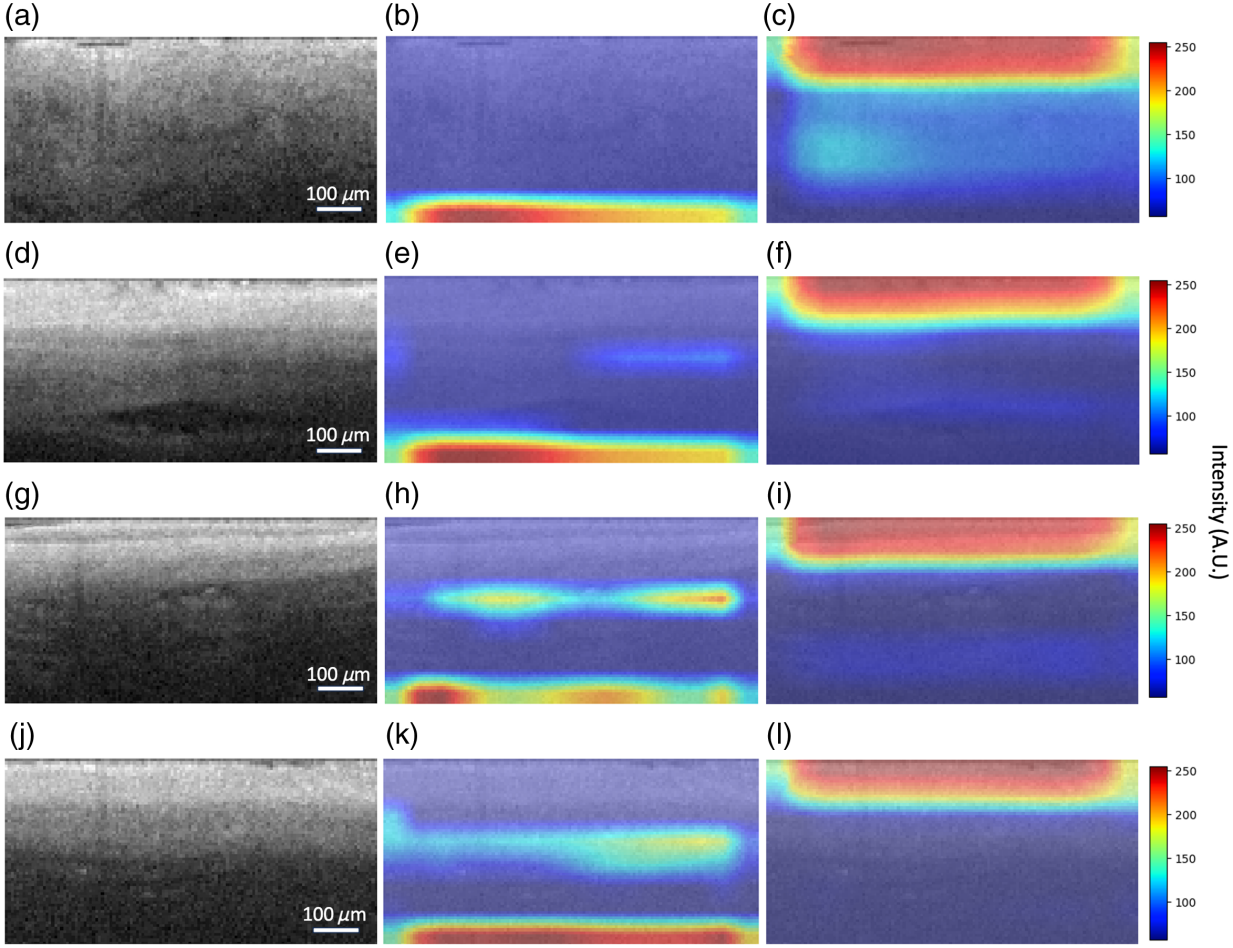
Sample correct classifications of OCT image patches from a region within the PV [(a), (d)] and a region within the LA [(g), (k)], and corresponding CNN GradCAM visualizations for the LA [(b), (e), (g), (k)] and PV [(c), (f), (i), (l)] gradients. The importance of pixels in the patch for class prediction is highlighted within these GradCAM maps. Higher-intensity pixels indicate regions with higher gradients, which are more impactful to classification than lower-intensity regions. For classifying the PV samples [(a), (d)], GradCAM shows that the PV gradient [(c), (e)] is sensitive to the top of the patch and notices the gradual intensity falloff. For classifying LA [(g), (j)], GradCAM shows that the LA gradient [(h), (k)] identifies the abrupt falloff of intensity at the endocardium-myocardium transition.

CNN performance was further examined by GradCAM (Fig. 8) to understand which regions of OCT image patches impacted the network’s decision-making the most. The LA to PV transition is characterized by a transition from endocardium and myocardium to venous media and adventitia, which have significant collagen content. This change is often accompanied by an increase in penetration depth in OCT images. For the endocardium to myocardium transition, a sharp intensity falloff is observed. Another trend is that a more heterogeneous image texture was typically seen in the pulmonary vein compared with the left atrium. In GradCAM analysis, the gradient for the PV label is most concerned with the top three of the images. For the LA label, the GradCAM shows significant attention to the middle third, particularly for correct classifications of the LA containing the high contrast transition from endocardium to myocardium. The network appears to identify the abrupt falloff in image intensity occurring at the transition from endocardium to myocardium.

### 3.3 Limitations and Future Work

This dataset within this study was imbalanced with the high-interest class, PV, being ∼22% of the patches. The primary reason for this is due to the variability of the dissections during the heart procurement process, and the amount of PV retained during the dissection process. In addition, the donors’ pathologies and medical history varied and impacted the amount of tissue remodeling present. This dataset contains OCT volumes of post-mortem human hearts from donors with varying disease histories (Table 1). Many of these diseases remodel the heart, causing the appearance of the PVs and LAs to vary across OCT volumes. Significant differences in classification performance across the seven cross-validation splits are likely due to this heterogeneity. Increasing the size of the dataset of this study will be key to further studies.

Ultimately, this work serves to advance substrate classification techniques for pulmonary vein isolation and ablation guidance that could be adapted for *in vivo* measurements. Performing this classification on OCT catheter data is required to evaluate the potential for clinical translation further. In this work, all analysis was performed on benchtop-imaged B-scans. Future work will include adapting the model to catheter based on *in vivo* OCT datasets. The collection of OCT image patches during ablation procedures does not incur significant imaging and computational overhead;^29^^–^^32^ this creates the potential for real-time substrate identification during ablation. By providing real-time structural information about the venoatrial junction, there is potential to improve the probability of a successful procedure and reduce complication rates of cardiac ablation.

## 4 Conclusion

To the authors’ knowledge, this is the first investigation showing automated classification of the LA and PV with OCT images of human venoatrial junctions. We have demonstrated classification using ML and DL techniques, namely, logistic regression and random forest, and three different CNN architectures. The DL approaches included a patch-based CNN, and two ensemble variations of the patch-based CNN in which the top statistically derived features were concatenated before the last dense activation. The ensemble deep learning methods performed the best.

## Data Availability

The data presented in this article are publicly available in the Columbia University Academic Commons at https://doi.org/10.7916/hrtt-xb60.
